# Clinical significance of incidental thyroid nodules identified on low-dose CT for lung cancer screening

**DOI:** 10.1186/2049-6958-8-56

**Published:** 2013-08-28

**Authors:** Jong Hoo Lee, Sun Young Jeong, Yee Hyung Kim

**Affiliations:** 1 Department of Pulmonary and Critical Care Medicine, Jeju National University Hospital, School of Medicine, Jeju National University, Jeju, Korea; 2 Department of Radiology, Jeju National University Hospital, School of Medicine, Jeju National University, Jeju, Korea; 3 Department of Pulmonary and Critical Care Medicine, Kyung Hee University Hospital at Gangdong, School of Medicine, Kyung Hee University, Seoul, Korea

**Keywords:** Lung cancer, Low dose CT, Screening, Thyroid cancer, Thyroid nodules

## Abstract

**Background:**

Incidental thyroid nodules (ITNs) are defined as newly encountered nodules identified on imaging performed for an unrelated purpose. In practice, ITNs are often detected on chest computed tomography (CT). We investigated the prevalence and clinical significance of ITNs detected on low-dose chest CT (LDCT) for lung cancer screening.

**Methods:**

We retrospectively reviewed the electronic medical records of patients with no history of thyroid disease who underwent LDCT for lung cancer screening between March 2009 and February 2012 at Jeju National University Hospital (Korea).

**Results:**

Among 1,941 patients that underwent LDCT, 55(2.8%) were found to have ITNs. Seven (12.7%) of those cases were malignant. The positive and negative predictive values of chest LDCT for the detection of incidental malignant thyroid nodules were 26.9% and 73.4%, respectively. Factors considered to be predictive of malignancy on LDCT were a mean attenuation value of 55 HU or more (p = 0.036) and the presence of dense calcifications (p = 0.048). Sex, age, location of the nodule, longest diameter of the lesion, AP/T (anteroposterior/transverse dimension) ratio, margins, density, presence of punctate calcifications, and thyroid enlargement had no significant predictive value in discriminating benign and malignant nodules. On multivariate analyses, a mean attenuation value above 55 was the only statistically significant feature (p = 0.048).

**Conclusions:**

A mean attenuation value greater than 55 HU on LDCT may be a useful predictive factor for differentiating malignant from benign lesions. Therefore, a careful assessment of the thyroid gland is necessary for patients undergoing LDCT for lung cancer screening.

## Background

The incidence and prevalence of thyroid cancer have been on the rise in recent years in Korea [1]. Although the reason for this increase is uncertain, the incidental detection of thyroid cancer through opportunistic screening may play a major role. As of 2012, the National Cancer Screening Program of Korea does not support routine screening for thyroid cancer [2]. Nevertheless, some patients have undergone screening for thyroid cancer at their own expense.

Incidental thyroid nodules (ITNs) are defined as newly encountered nodules identified on imaging such as ultrasonography (US), computed tomography (CT), or magnetic resonance (MR) imaging performed for an unrelated purpose. In the general population, the prevalence of thyroid nodules is very high [3]. Therefore, the incidence of ITNs is expected to increase along with increased screening for a variety of diseases as well as technical advances in the various imaging modalities [4,5].

Since the recent publication of The National Lung Screening Trial (NLST) [6], an increase in low-dose chest CT (LDCT) for routine lung cancer screening in patients at high risk for the disease is expected. In practice, thyroid nodules are commonly detected on chest CT studies performed to evaluate non-thyroid diseases [7]. There have been few reports, however, that have examined ITNs using LDCT intended for lung cancer screening. Moreover, there is a paucity of data regarding whether lesion characteristics on LDCT can help to distinguish benign from malignant thyroid nodules.

In this study, we aimed to determine the prevalence of ITNs as well as rates of malignancy in patients with no history of thyroid disease that underwent LDCT for lung cancer screening. In addition, features that could be helpful in differentiating between benign and malignant thyroid nodules on LDCT were evaluated.

## Methods

### Study population

Currently there is no official guideline for lung cancer screening in the Korean population. Therefore, criteria for identifying the at-risk population that requires lung cancer screening are not established yet. The health promotion center of Jeju National University Hospital has performed LDCT for lung cancer screening since March 2009 based on the results of previous studies including the NLST. The main indications for screening include smoking history, family history of lung cancer, and concern for lung cancer in subjects without a smoking history or positive family history (voluntary testing).

We retrospectively reviewed the electronic medical records of all patients that underwent LDCT for lung cancer screening between March 2009 and February 2012 at Jeju National University Hospital. In this 540-bed secondary teaching hospital, 126 patients with thyroid cancer underwent surgical resection in 2011. All subjects were asymptomatic and had had no history of cancer within the past five years. We evaluated the medical records of each patient and the histopathologic results of the thyroid lesions identified on LDCT. This study protocol was approved by the Ethical Review Committee. Informed consent was waived due to the retrospective nature of the study.

### Analysis of LDCT features

All CT examinations were performed using a 16-detector row CT scanner (Sensation 16; Siemens, Erlangen, Germany) or a dual-source CT scanner (SOMATOM Definition, Siemens Medical Solutions, Forchhein, Germany). CT images were obtained from 3 cm above the sternal notch to the middle of both kidneys without contrast enhancement. The entire thyroid gland was included on all imaging studies. The acquisition parameters were 120 kVp, a pitch of 1.375, a 0.5 second gantry rotation time, a 40 mm beam width, and 25–40 mAs with adjustment of the automatic exposure control. The axial images were reconstructed with a 2 mm slice thickness using both the bone and standard algorithms. A single radiologist with seven years of experience in chest CT scan interpretation reviewed all the CT images.

An ITN was defined as a new asymptomatic thyroid nodule that was discovered on LDCT performed to evaluate a disease unrelated to the thyroid gland. A benign thyroid nodule was defined as follows: 1) a benign lesion such as an adenomatous hyperplasia or follicular nodule that was histologically confirmed through fine needle aspiration (FNA) or surgical resection, or 2) a lesion with US findings characterized predominantly by cystic lesions with small solid components. In contrast, a malignant thyroid nodule was defined as a nodule that was histologically confirmed as cancerous via FNA or surgical resection.

The following parameters were evaluated on LDCT for each patient: 1) focal or multiple nodules, 2) location of the dominant nodule, 3) longest diameter of the lesion, 4) ratio of the anteroposterior dimension to the transverse dimension (AP/T ratio), 5) mean attenuation value (Hounsfield units, HU), 6) margins (well circumscribed versus ill defined), 7) density of the nodule (hypodense, isodense, hyperdense, heterogeneous, ordensely calcified), 8) calcification (densely calcified, punctate calcification, or rim calcification), and 9) presence of thyroid enlargement.

Based upon the above parameters, we investigated clinical and radiological features of all patients in whom ITNs were detected on LDCT. We also analyzed clinical and radiological differences between benign and malignant thyroid nodules.

### Statistical analyses

Data are presented as numbers (%) or medians (interquartile range, IQR) unless otherwise stated. Continuous variables were compared using Student’s t-tests for normally distributed variables and Mann–Whitney *U*-tests for non-normally distributed variables. Univariate analysis was carried out using Chi-square tests or Fisher’s exact tests for categorical data. Multivariate analysis was performed using logistic regression analysis. P < 0.05 was considered to be statistically significant. A receiver-operating-characteristic (ROC) curve analysis was done to measure and compare the accuracy of LDCT parameters in differentiating benign from malignant thyroid nodules. The diagnostic accuracies of various LDCT parameters were expressed as the area under the corresponding ROC curve (AUC). All analyses were performed using SPSS version 14.0 (SPSS Inc., Chicago, IL, USA).

## Results

### Study enrollment and final diagnoses

**Figure 1:**
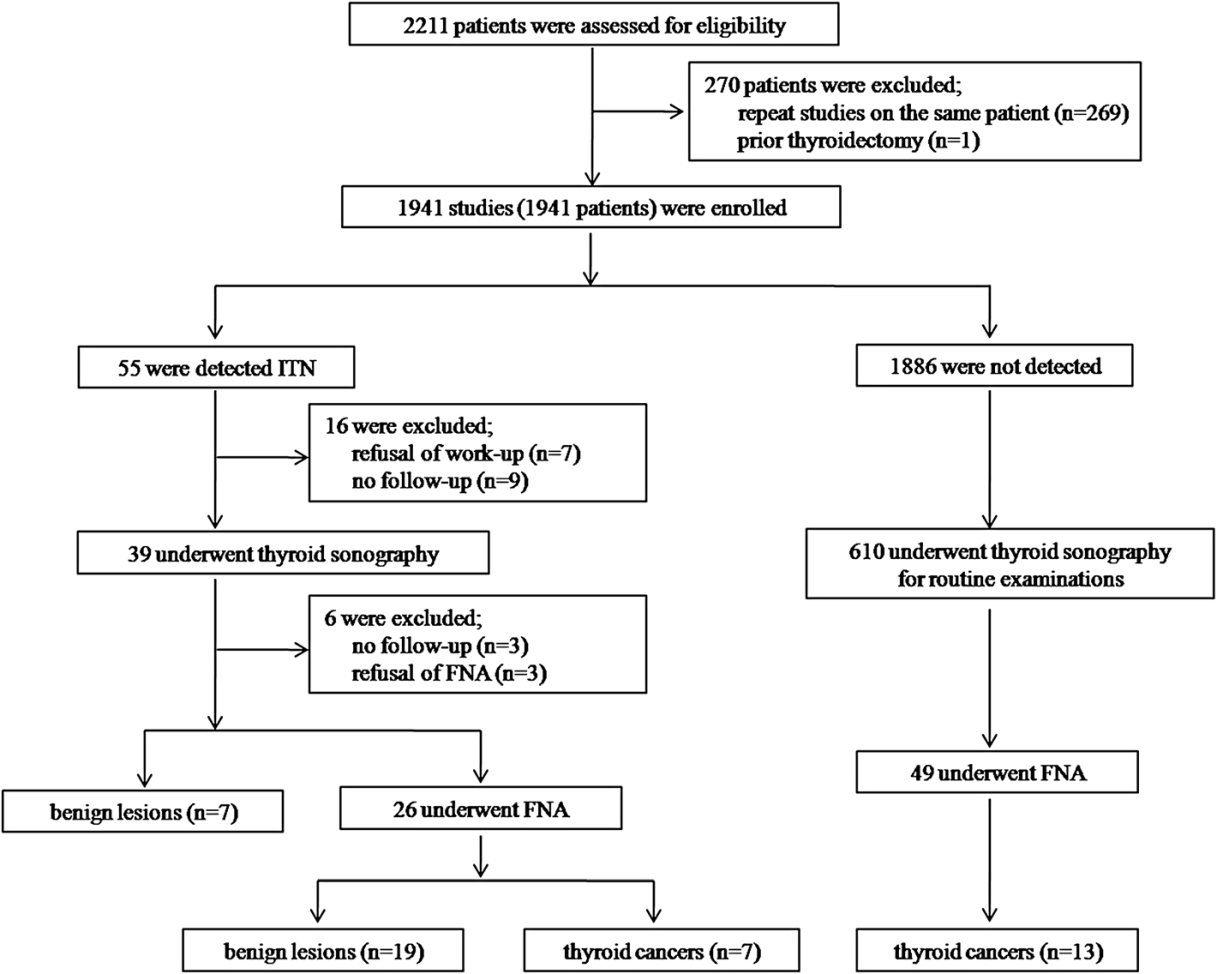
**Patient enrollment and outcomes.** FNA, fine needle aspiration; ITN, incidental thyroid nodule(s).

Figure 1 shows patient enrollment data and final diagnoses. 270 out of 2,211 LDCT scans were excluded from this study because they were repeated on the same patient. One LDCT study was also excluded because of prior thyroidectomy. The remaining 1,941 patients were included in this study, 1,300 out of whom were men and 641women. The median age of the study population was 53 years (IQR: 43 to 63).

**Figure 2:**
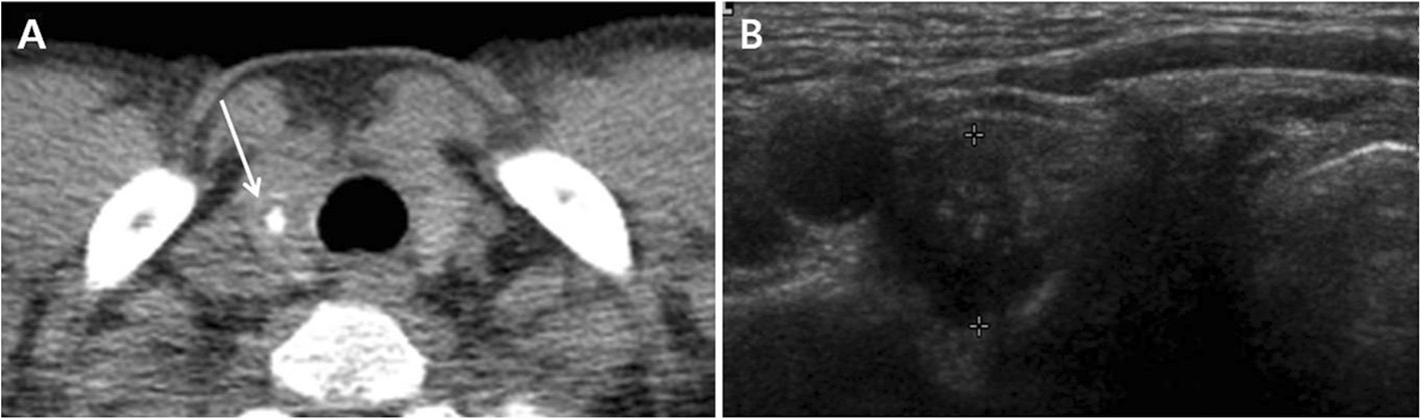
**A 60**-**year**-**old man with papillary thyroid carcinoma. (A)** Low-dose helical CT scan demonstrated a 6 mm densely calcified nodule (arrow) in the right thyroid lobe. **(B)** Transverse US image of the right thyroid lobe showed a hypoechoic mass (calipers) with internal calcifications.

**Figure 3:**
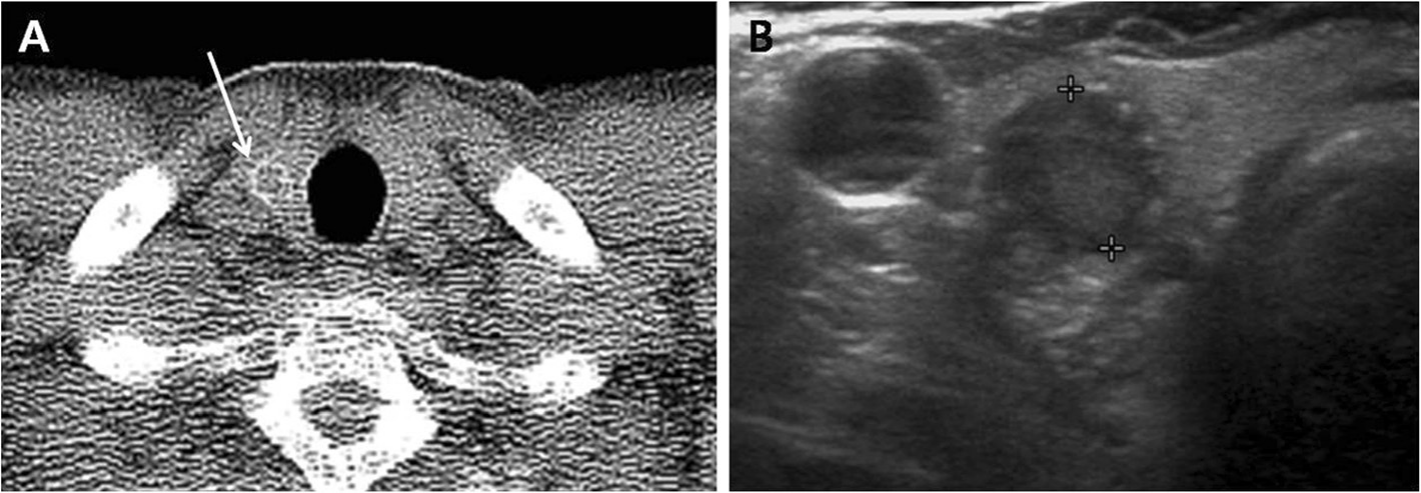
**A 53**-**year**-**old man with papillary thyroid carcinoma. (A)** Low-dose helical CT scan demonstrated a 9 mm hypodense nodule (arrow) with peripheral rim calcification in the right thyroid lobe. **(B)** Transverse US image of the right thyroid lobe showed a hypoechoicnodule (calipers) with peripheral rim calcification.

**Figure 4:**
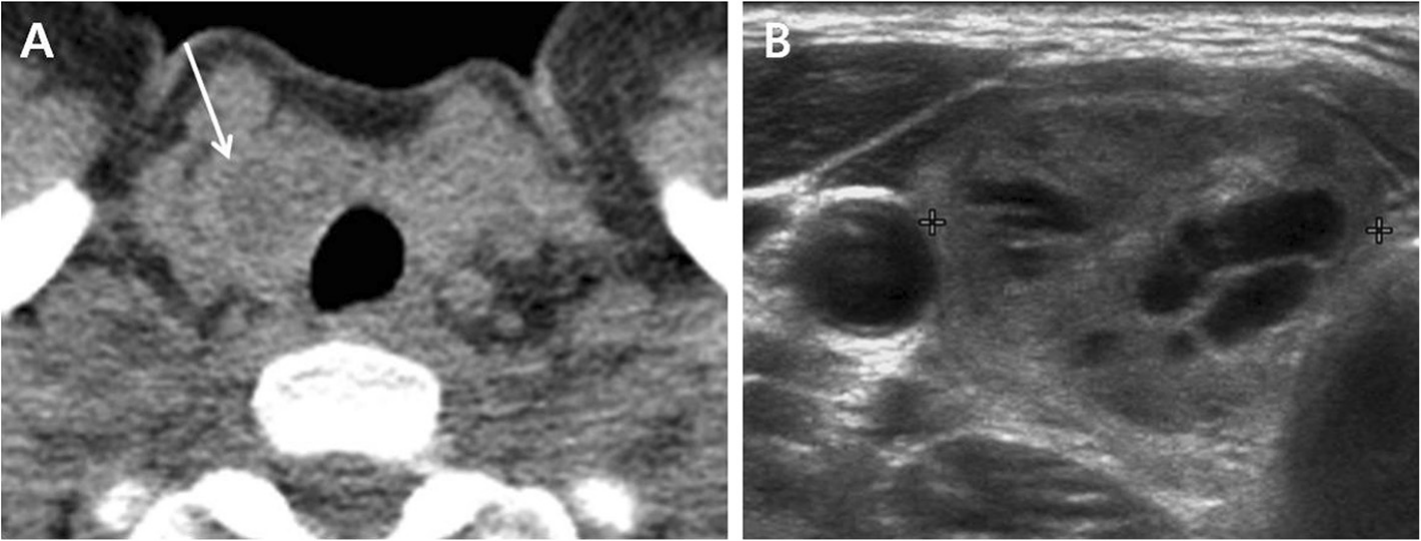
**A 49-year-old woman with an adenomatous goiter. (A)** Low-dose helical CT scan demonstrated a 21 mm hypodense nodule (arrow) without calcification in the right thyroid lobe. **(B)** Transverse US image of the right thyroid lobe showed a mixed solid and cystic nodule (calipers).

Fifty-five (2.8%) out of 1,941 patients were found to have ITNs on LDCT. Among them, 16 were excluded due to refusal of further work-up (n = 7) or were lost to follow up (n = 9). The remaining 39 patients underwent subsequent thyroid US. Of them, 7 patients were found to have benign lesions that did not require FNA (radiological confirmation). Twenty-six patients underwent FNA, which revealed 19 benign nodules and 7 malignant nodules (histological confirmation). As a result, 7(12.7%) malignant nodules were confirmed in 55 patients with ITNs detected on LDCT screening, all of which were identified as papillary carcinoma. Two patients underwent total thyroidectomy and one refused surgical resection. The remaining 4 were referred to other hospitals or lost to follow up. Figures 2, 3 and 4 show the features seen on LDCT and the corresponding images on US in patients with confirmed benign or malignant thyroid nodules.

Out of the 1,886 patients whose thyroid nodules were not observed on LDCT, 49 patients underwent FNA due to nodules detected on US. Among them, thirteen (26.5%) were found to have malignant thyroid nodules. The sensitivity, specificity, false positivity, false negativity, positive predictive value, and negative predictive value of LDCT for detection of malignant thyroid nodules were 35.0%, 65.4%, 34.5%, 65.0%, 26.9%, and 73.4%, respectively (Table 1).

**Table 1:** **Sensitivity**, **specificity**, **false positivity**, **false negativity**, **positive predictive value**, **and negative predictive value of incidental thyroid nodules detected on low**-**dose helical CT in patients undergoing fine needle aspiration for histologic confirmation**

		**Thyroid cancer**	
		**Present**	**Absent or undetermined**
Low-dose chest CT	Positive	7	19
	Negative	13	36

Sensitivity = 35.0%, Specificity = 65.4%, False positivity = 34.5%, False negativity = 65.0%, Positive predictive value = 26.9%, Negative predictive value = 73.4%.

### Clinical characteristics and low-dose CT features of patients with ITNs

Table 2 shows the clinical characteristics and LDCT features of patients with ITNs. A total of 72 nodules were identified in55 patients. ITNs were more common in women, and the median age of these patients was 58 years (IQR: 53–68).

**Table 2:** **Baseline characteristics of patients and low**-**dose CT features of incidental thyroid nodules**

**Clinical characteristics**	
Sex (men)	21 (38.1%)
Age (years)	58 (53–68)
≥ 60 years	26 (47.2%)
Low-dose CT features	
Lesions	
Focal	44 (80%)
Multiple	11 (20%)
Number of nodules	72
Location	
Right	43 (59.7%)
Left	25 (34.7%)
Isthmus	4 (5.5%)
Longest diameter (mm)	9.1 (6.2–15.6)
≥10 mm	31 (43.1%)
AP/T ratio^†^	1.1 (0.9–1.2)
AP/T ratio ≥ 1	51 (70.8%)
Mean attenuation value (HU)	54 (33–114)
Margin	
Well- circumscribed	42 (58.3%)
Ill-defined	30 (41.7%)
Density	
Hypodense	50 (69.4%)
Isodense	0 (0%)
Hyperdense	1 (1.3%)
Heterogenous	2 (2.7%)
Densely calcified	19 (26.3%)
Any calcifications	26 (36.1%)
Rimcalcification	4 (5.5%)
Punctate calcification	4 (5.5%)
Thyroid enlargement	11 (15.2%)

Data are presented as numbers (%) or medians (interquartile range, IQR).

^**†**^The ratio of the anteroposterior dimension to the transverse dimension of the nodule.

Most patients (80%) had focal thyroid nodules. The number of nodules in patients with multiple lesions ranged from 2 to 5. The location of thyroid nodules was frequently the right side of the thyroid gland (59.7%). The median longest diameter, AP/T ratio, and mean attenuation value were 9.1 mm (IQR: 6.2–15.6), 1.1 (IQR: 0.9–1.2), and 54 HU (IQR: 33–114), respectively. The margins of the nodules were frequently well-circumscribed (58.3%) and most were hypodense (69.4%). In addition, there were 26 nodules (36.1%) with calcifications.

### Differences in clinical characteristics and low-dose CT features of benign and malignant thyroid nodules

We analyzed the differences in clinical characteristics and LDCT features between benign and malignant thyroid nodules. Twenty-six patients who had a final diagnosis of benign thyroid nodules had a total of 39 nodules. Seven patients who were found to have malignant nodules had a total of 11 nodules.

On univariate analyses, factors that distinguished malignant thyroid nodules from the benign ones included a mean attenuation value of 55 HU or more (p = 0.036) and the presence of dense calcifications (p = 0.048) (Table 3). Sex, age, location of the nodule, longest diameter of the lesion, AP/T ratio, margins, density, presence or pattern of calcifications, rim calcifications, and thyroid enlargement were not significantly different between benign and malignant thyroid nodules. Multivariate analysis showed that a mean attenuation value of 55 HU or more was statistically significant (p = 0.048, Table 4).

**Table 3:** **Differences in clinical characteristics and low**-**dose CT features between benign and malignant thyroid nodules**

**Factor**	**Benign (n = 39)**	**Malignant (n = 11)**	***p***
Sex (men)	18 (46.1%)	8 (72.7%)	0.119
Age (≥ 60 years)	15 (38.4%)	7 (63.6%)	0.178
Location of nodule			
Right	22 (56.4%)	6 (54.5%)	1.000
Left	15 (38.4%)	5 (45.4%)	0.736
Isthmus	2 (5.1%)	0 (0%)	1.000
Longest diameter (≥ 10 mm)	18 (46.1%)	3 (27.2%)	0.319
AP/T ratio^†^(≥1)	27 (69.2%)	6 (54.5%)	0.475
Mean attenuation value (≥ 55 HU)	3 (27.2%)	8 (72.7%)	0.036
Ill-defined margin	18 (46.1%)	2 (18.1%)	0.163
Density			
Hypodense	32 (82.0%)	6 (54.5%)	0.105
Isodense	0 (0%)	0 (0%)	NA^*^
Hyperdense	0 (0%)	0 (0%)	NA
Heterogeneous	1 (2.5%)	0 (0%)	1.000
Densely calcified	6 (15.3%)	5 (45.4%)	0.048
Any calcifications	10 (25.6%)	6 (54.5%)	0.140
Punctate calcification	3 (7.6%)	0 (0%)	1.000
Rim calcification	1 (2.5%)	1 (9.0%)	0.395
Thyroid enlargement	8 (20.5%)	0 (0%)	0.174

Data are presented as numbers (%).

^†^The ratio of the anteroposterior dimension to the transverse dimension of the nodule.

^*^Not available.

**Table 4:** Multivariate analysis for detection of malignant thyroid nodules on low-dose CT

**Variables**	**Odds ratio**	**95% Confidence interval**	**p**
Male	3.626	0.638-20.597	0.146
Mean attenuation value ≥55 HU	9.970	1.023-97.179	0.048
AP/T ratio^†^ ≥ 1	0.387	0.074-2.026	0.261
Longest diameter ≥ 10 mm	1.207	0.114-12.820	0.876
Densely calcified	0.884	0.088-8.859	0.917
Ill-defined margin	0.214	0.025-1.798	0.155

^†^The ratio of the anteroposterior dimension to the transverse dimension of the nodule.

**Figure 5:**
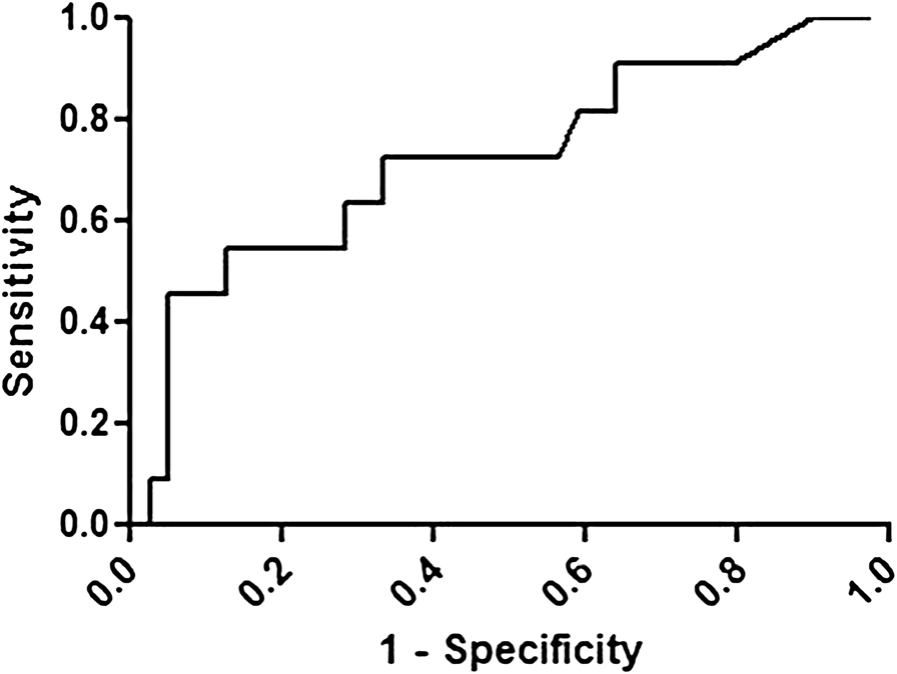
**ROC curves of low**-**dose CT when a mean attenuation value of 55 HU was used to differentiate benign from malignant thyroid nodules.**

When only the mean attenuation value was used to differentiate benign from malignant thyroid nodules on ROC curve analysis, the area under the curve (AUC) of LDCT was 0.7238 ± 0.0920 (p = 0.024, Figure 5). Using the ROC curve analysis, the cut-off attenuation value that avoids missing the most malignant thyroid nodules was 54.5 HU, and its sensitivity and specificity were 72.7% and 66.6%, respectively.

## Discussion

Thyroid nodules are commonly encountered in clinical practice. However, the prevalence of thyroid nodules largely depends on the method of screening and the population being evaluated. Autopsy data provides the gold standard for determining the true prevalence of thyroid nodules, with previous autopsy studies reporting a prevalence of 8.2 to 64.6% [3,8,9]. On the other hand several reports based on US data have shown a prevalence of 19–46% [5,10-12]. The risk that these nodules will be found malignant is relatively low, ranging from 1.5 to 17% [7]. Recently, the NLST Research Team published its results showing that lung cancer screening using chest LDCT reduced lung cancer-related mortality by 20% [6]. As a result, the use of LDCT for lung cancer screening is expected to increase rapidly. As chest LDCT is becoming a much more common imaging modality, the incidence of thyroid nodules detected incidentally is also expected to increase. This may in turn lead to an increase in the number of detected malignant nodules.

Few studies have evaluated the role of LDCT in the detection of extra-pulmonary malignancies in a population at high-risk for lung cancer [13-18]. In a retrospective study of 5,201 patients who underwent LDCT for lung cancer screening, 27(0.5%) extra-pulmonary malignancies were incidentally diagnosed, 3(0.05%) out of which were thyroid cancers [15]. In another study, a total of 6 malignancies were found over the course of a 5-year lung cancer screening program. However, no thyroid cancer was found in this endemic area, despite a high number of ITNs [13]. Our single-center, LDCT-based study found that thyroid nodules were incidentally detected on LDCT in 2.8% (55/1941) of all subjects with no previous evidence of thyroid disease, and 12.7% of these were found to be malignant. The difference in detection rates between our study and previous studies may in part be due to the population evaluated.

In contrast, studies based on contrast-enhanced CT showed a higher prevalence of ITNs. One study reported the prevalence rate of ITNs to be 15.6% using contrast-enhanced neck or chest CT, and at least 11.3% of these were malignant or potentially malignant lesions [19]. Additionally, Yoon et al. conducted a study using 16-MDCT contrast-enhanced CT of the neck in 734 patients without previous thyroid disease [20]. They reported that 16.8% of subjects had ITNs and 9% of these were malignant nodules. A lower detection rate of ITNs using LDCT compared with contrast-enhanced CT may be due to the limitations of interpreting the images without contrast. In addition, characterization of nodules less than 1 cm in size would not be appropriate since LDCT is typically performed using a 5 mm slice thickness.

To date, US has generally been the preferred imaging modality for the evaluation of thyroid nodules. Therefore, US features associated with a higher risk of malignancy are relatively well established, and include the presence of fine or coarse calcifications, hypoechogenicity, irregular margins, absence of a halo, predominantly solid composition, lesion shape that is taller than wide, and internal vascular flow [20]. However, LDCT features indicative of malignancy are not established yet, since LDCT is generally not used to evaluate thyroid nodules. In this study, we tried to identify ITNs features that are predictive of malignancy on LDCT. A previous CT-based study found that the diagnostic imaging features related to malignancy included nodular or rim calcifications, an AP/T ratio greater than 1.0, and a mean attenuation value greater than 130 HU [7]. Another study reported that CT could not reliably differentiate features of benign from malignant thyroid lesions [19]. In our study, the factors that were predictive of malignancy in thyroid nodules were a mean attenuation value of > 55 HU and the presence of dense calcifications. On multivariate analysis, only a mean attenuation value of 55 HU exhibited statistical significance.

Despite this significant difference in mean attenuation values between benign and malignant nodules, it can be difficult to distinguish these lesions based on the mean attenuation value alone. When the mean attenuation value was applied in this study, the AUC of ROC curves was 0.7238 ± 0.0920 (Figure 5). This indicates that the mean attenuation value has relatively low sensitivity (72.7%) and specificity (66.6%), and thus a significant overlap in mean attenuation values may exist between benign and malignant nodules.

As mentioned above, use of LDCT for lung cancer screening will lead to increase in the number of ITNs detected. As a result, it will be necessary to develop guidelines for how to manage incidentally detected malignant nodules. The management of ITNs has been controversial, and despite an increase in the frequency of surgeries to treat small thyroid cancers, thyroid cancer-related mortality has not improved [21]. In addition, it was reported that more than 70% of tumors in patients with papillary microcarcinoma that were followed without surgical intervention remained stable or decreased in size, even after > 5 years [22]. This indicates that overdiagnosis by LDCT can lead to overtreatment in healthy persons with ITNs, thereby increasing medical costs. Interestingly, malignant nodules histologically confirmed in our study were all papillary carcinoma.

We are aware this study has several limitations. Firstly, we were unable to determine accurate indications for screening by reviewing the medical records of individual subjects. The criteria for at-risk patients who would benefit from lung cancer screening have not been established yet in Korea. Therefore, it is likely that a percentage of the study population is not truly at-risk for malignancy along with those that received voluntary testing. As a result, our cohort of patients may be different from those used in previous LDCT-based studies. Secondly, we were unable to obtain data regarding smoking status at the time of the study. Since most cancers are related to smoking, it is important to know how many smokers were included in the study population. Additionally, there may be radiological features of ITNs that are associated with smoking history.

## Conclusions

In conclusion, although thyroid nodules on LDCT are detected incidentally in a small percentage of the population, some of these lesions may be malignant. Therefore, careful observation of the thyroid gland as well as other intra-thoracic organs is required when evaluating LDCT. A high mean attenuation value of 55 HU or more, in particular, rather than margins, size, calcification, or density, may be a useful predictive factor for differentiating malignant from benign lesions.

## Competing interests

The authors have reported no significant conflicts of interest with any companies/organizations whose products or services may be discussed in this manuscript.

## Authors’ contributions

JHL contributed to the conception and design of the study and drafted the original manuscript. JHL and SYJ collected clinical and radiological data and performed the statistical analysis. YHK was involved in the interpretation of data, review of the study, and final revision of the original manuscript. All authors read and approved the final manuscript.
